# The impact of the framing effect on performance in a simulated emergency anaesthetic scenario in medical officers: A prospective, randomised, double-blinded study

**DOI:** 10.4102/jcmsa.v3i1.262

**Published:** 2025-12-12

**Authors:** Guido Ferreira, David G. Bishop, Pragasan D. Gopalan

**Affiliations:** 1 Discipline of Anaesthesiology, Pain Medicine & Critical Care, University of KwaZulu-Natal, Pietermaritzburg, South Africa

**Keywords:** cognitive errors, framing effect, anaesthesia, simulation, clinical reasoning

## Abstract

**Background:**

Cognitive errors in anaesthesia may contribute significantly to medical error in the perioperative environment, but few studies have been conducted in this area. The framing effect is a cognitive bias that occurs when a problem is presented in different ways, potentially leading to changes in clinical decision-making.

**Methods:**

The authors conducted a single-centre, prospective, randomised, double-blinded study with the aim of determining the impact of the framing effect in medical officers who have recently passed their Diploma in Anaesthesia examination in the Pietermaritzburg Anaesthetic Department. All participants underwent the same simulated emergency scenario related to high airway pressures (because of a bronchial plug) in a ventilated patient under anaesthesia. Participants were allocated either to a control group (receiving a neutral handover) or to an experiment group (receiving a handover that included additional information relating to asthma). The authors also collected quantitative data related to clinical performance and qualitative data related to participants’ experience of the scenario.

**Results:**

The study included a total of 34 medical officers, with 17 in each group. There was no difference in median times to diagnosis (control group 240 [interquartile range {IQR} 195–240] vs experimental group 240 [IQR 162−240] s; z = −0.433, *p* = 0.6648). There were no differences in secondary outcomes. Participants reported a positive learning experience that may influence future training methods.

**Conclusion:**

The study was unable to demonstrate objective evidence for the framing effect in this simulation study. Future studies can use these findings to perform sample size calculations for larger studies to investigate this important area.

**Contribution:**

This study offers insight into the relationship between cognitive bias and clinical decision-making within anaesthesia simulation training. The findings contribute to a better understanding of how framing effects may influence trainee responses in simulated clinical scenarios. This work aligns with the JCMSA’s focus on medical education and training.

## Introduction

Medical error remains a common and important problem, with a significant impact on healthcare outcomes. Approximately 1 in 20 patients is exposed to preventable harm,^1^ and human error accounts for up to 87% of all medical errors, with varying prevalence stratified by specialty and clinical situation.^2^ There is a lack of good data concerning medical errors in the South African context, but the medical error rate in developing countries has been estimated to occur in approximately 1 in 12 patients.^3^ Cognitive errors are thought-process errors or thinking mistakes, which lead to incorrect diagnoses, treatment or both.^4^ Cognitive errors in anaesthesia likely contribute significantly to medical error in the perioperative environment, but this is an understudied area. The framing effect is one example of a cognitive bias that can have an impact on medical decision-making. A framing error occurs when subsequent thinking is swayed by the leading aspect of the initial presentation.^4^ This implies that time-sensitive decisions during emergencies might be influenced by the way in which medical information is framed.^5^ To our knowledge, no studies examining the potential impact of the framing effect in anaesthetic medical officers in an emergency scenario have been conducted.

The purpose of this study was to compare the impact of the framing effect on clinical performance in an emergency simulated scenario under two different framing conditions (Control vs Experiment). We hypothesised that framing the patient as an asthmatic would bias participants towards bronchospasm as the primary diagnosis. This anchoring and/or fixating was expected to delay consideration of alternative causes, such as a mucous plug, thereby prolonging the time to correct diagnosis and initiation of appropriate corrective action. The way a clinical simulation scenario is framed will create a significant and clinically relevant bias, resulting in a prolonged time to diagnosis and/or implementation of corrective measures. We further aimed to assess the participants’ perception of the simulation, their performance and their critical thinking.

## Research methods and design

We conducted a prospective, randomised, double-blinded study on medical officers who had recently passed their Diploma in Anaesthesia (DA) examination, in the Pietermaritzburg Complex (KwaZulu-Natal, South Africa). Ethical approval was obtained from the KwaZulu-Natal Department of Health (KZ_202110_028) and the University of KwaZulu-Natal Biomedical Research Ethics Committee (BREC/00003229/2021). Informed, written consent was obtained from all participants. The study took place at the Grey’s Hospital Simulation Centre.

### Participants

We included anaesthetic medical officers who had recently passed their DA examination in the Pietermaritzburg Complex and were agreeable to participation. We aimed to exclude anyone with prior knowledge of the scenario or with more than 2 years of experience post their DA examination. This was to ensure a more homogenous study population in terms of experience.

### Procedure

Participants were randomised to either the control or experimental group. Randomisation occurred through sealed, opaque envelopes containing the scenario briefing. Both participants and examiners were blinded to group allocation, and examiners were not aware of the study aims or primary outcome. There were two examiners for the duration of the study. They were told that they were involved in the assessment of a simulated emergency scenario. The examiners were briefed on the nature of the emergency scenario and were also familiarised with the assessment sheet. They were required to record the time at which various actions or responses occurred in the participants. They assessed the candidates separately on standardised assessment sheets and were blinded to each other’s assessment. The assessment sheet and the outline of the simulation can be found in Online Appendix 1.

Candidates were assigned a study number on commencement. They were instructed not to discuss the details of the study with anyone. To limit the impact on service delivery and time spent being assessed and to ensure consistent, standardised briefing, both the introduction and debriefing occurred with prerecorded video that participants watched before and after the simulation. Participants watched the briefing individually before proceeding with the simulation. Candidates were asked to verbalise their thought processes during the scenario.

The scenarios in the envelope differed only in the framing of the initial clinical presentation. Debriefing occurred with a prerecorded video that participants watched at the end of the scenario. It aimed to provide individuals with the necessary knowledge to handle similar scenarios in future clinical practice. The aim of the debriefing was also to reassure candidates, provide any emotional support and inform them that once the study has been completed, the results and relevant literature will be emailed to all the participants. If candidates required further debriefing, contact details of the principal investigator were made available for the candidates to discuss any concerns that arose during the simulation.

We further collected qualitative data to assess participant experience during and after the simulation through a structured questionnaire. We used a rank-order response scale for questions not requiring a written answer.

### The simulation

The study was conducted in the Grey’s Hospital Simulation Centre. We made use of a high-fidelity human patient simulator (Stan model, Human Patient Simulator™; Medical Education Technologies, Inc. (METI), Sarasota, FL, USA), controlled using Müse^®^ instructor workstation software (METI/CAE Healthcare, Sarasota, FL, USA), was used for all simulation scenarios. Participants had access to waveform capnography, invasive arterial pressure (ART) monitoring, pulse oximetry and electrocardiogram (ECG) monitoring. Additional equipment available during the simulation included a Dräger Fabius^®^ anaesthesia workstation (Drägerwerk AG & Co. KGaA, Lübeck, Germany) anaesthetic machine, airway management equipment and surgical drapes.

In the briefing video, candidates were told that they would be going through a simulation of a clinical scenario that was outlined through written instructions. Participants would be taking over the care of an anaesthetised patient from the attending doctor. The written handover was provided in identical opaque envelopes, mixed in a box, with an even number of experimental and control scenarios. Participants individually selected an envelope from the box.

The scenario involved an intubated patient, who became progressively hypoxic during the simulation, commencing at 120 s. The cause of the hypoxia was obstruction of the endotracheal tube by a dislodged mucous plug. Clinical signs included a silent chest on auscultation and high airway pressures on ventilation. The differential diagnosis provided a clinical conundrum for participants, as this could also be because of several possible causes, including bronchospasm. The initial handover described the patient as previously healthy (control group) or as a poorly controlled asthmatic (experimental group).

There were no spontaneous examiner prompts during the simulation to participants. However, the examiner was permitted to provide relevant information linked to participant actions. For example, should the participant attempt suctioning with a flexible suction catheter, the examiner was permitted to inform the candidate that there was resistance to passing the catheter. The simulation ended after correct diagnosis and corrective action, or at a time of 360 s when the patient develops pulseless electrical activity (PEA), and CPR is commenced. Debriefing took place shortly after the simulation.

The aim of the study was to determine the impact of the framing effect on an emergency simulation scenario in a simulated theatre environment. The primary outcome was the time to diagnosis, measured from the commencement of oxygen desaturation at 120 s to the verbalisation of the diagnosis. We collected relevant secondary outcomes linked to relevant time-sensitive decisions.

### Statistical analysis

We assessed the normality of data using histograms and measures of skewness and kurtosis. For continuous outcomes, we used median and range to describe the data, and comparisons between groups were made with the Wilcoxon Mann-Whitney test. Categorical data were expressed as count (percentage), and comparisons between groups were made using the Chi-squared test. For the sample size calculation, we assumed an average time to diagnosis of 120 s (following handover at 120 s) in the control group. To achieve a power of 80% and a level of significance of 5% (two sided), to detect a true difference of 15 s, we required a total of 34 subjects (17 per group). It was anticipated that there would be approximately 10 available candidates per exam, with an overall total of 30 participants (15 per group). We anticipated getting the required number within 18 months of data collection. Statistical analysis was performed using Stata Release 15.1^©^ (StataCorp LP, College Station, Texas, US).

### Ethical considerations

Ethical clearance to conduct this study was obtained from the KwaZulu-Natal Department of Health (KZ_202110_028) and the University of KwaZulu-Natal Biomedical Research Ethics Committee (BREC/00003229/2021). Informed, written consent was obtained from all participants. All procedures were in accordance with the ethical standards of the responsible committee on human experimentation (institutional and national) and with the Helsinki Declaration of 1975, as revised in 2008.

The authors declare that this submission is in accordance with the principles laid down by the Responsible Research Publication Position Statements as developed at the 2nd World Conference on Research Integrity in Singapore, 2010.

## Results

**FIGURE 1:**
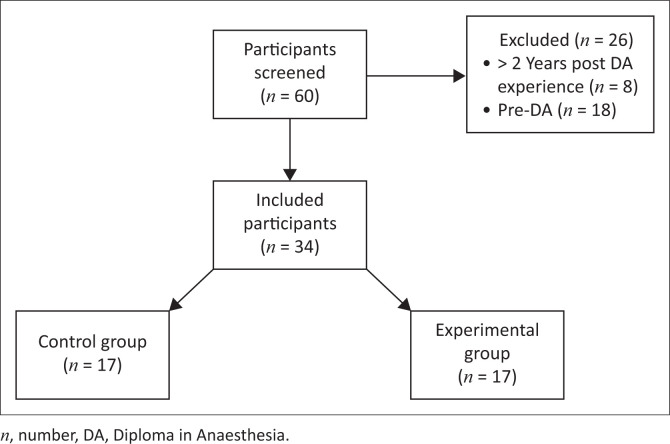
Participant flow diagram.

The participant recruitment process is illustrated in Figure 1. The final analysis included 34 participants, with 17 in each group. No eligible participant refused consent.

The primary outcome was the difference in time to correct diagnosis (bronchial plug). There was no difference in median times (control group 240 [interquartile range {IQR} 195–240] vs experimental group 240 [IQR 162–240] s; *z* = −0.433, *p* = 0.6648). Further action-based secondary outcomes are shown in Table 1.

**TABLE 1:** Secondary outcomes.

Endpoint	Control		Experimental		*z*-score	*p*-value
	Median	IQR	Median	IQR		
Increased oxygen	22.5	12–75	20	11–24	−0.96	0.34
Auscultation	49.3	34–71	30.5	21–57	−1.43	0.15
Assessed DOPE	38.3	15–60	45	25–70	0.37	0.71
Confirmed ETT	107.5	75–130	80	68–123	−0.74	0.46
Suctioned ETT	72.5	60–158	143.75	95–168	1.41	0.16
Removed ETT	146.3	120–195	150	120–172	−0.36	0.72

IQR, interquartile range; DOPES, dislodged tube (auscultation), obstruction (foreign body, mucous plug, secretions), pneumothorax, equipment failure, stacking (bronchospasm); ETT, endotracheal tube.

Secondary outcomes relating to diagnostic reasoning were as follows: Participants in the control group entertained the correct diagnosis in 12/17 (70.6%) vs 9/17 (52.9%) in the experimental group (*p* = 0.48). In the control group, only 1/17 (5.9%) participants were unable to entertain more than one diagnosis, versus 3/17 (17.6%) in the experimental group (*p* = 0.60).

We used a questionnaire with a rank-order (Likert) response scale for questions not requiring a written answer (1: Bad, 5: Excellent). The participants ranked the quality of the briefing (5, IQR 4–5) and the relevance of the scenario (5, IQR 5–5) as excellent. The quality of the simulation was ranked as 4.5 (IQR 4–5) and the realism of the simulation as a 4 (IQR 4–5). The participants’ experience of the simulation was ranked lower, at 3.5 (IQR 3–4).

We also asked participants to state their leading diagnosis after the scenario: bronchospasm was the diagnosis in 9/17 (52.9%) of the experimental group vs 8/17 (47.1%) in the control group. We further asked if candidates felt that the handover had been problematic in any way, and 12/17 (70.6%) in the experimental group vs 6/17 (35.3%) in the control group felt misled by the scenario.

## Discussion

Our study showed that the framing of a clinical handover did not result in a difference in time to correct diagnosis in a simulated emergency scenario. Despite the cognitive trap, there was very little difference in performance between the two groups. There were also no significant differences in secondary outcomes between groups, which would require a larger study. Both participant and examiner feedback were positive with regard to relevance, realism and quality, and simulation is likely to become a part of the routine DA curriculum.

The psychology of decision-making is becoming increasingly appreciated in the non-medical and medical literature.^4^ Cognitive biases like framing can affect subsequent thinking. The high prevalence of human error in anaesthesia is likely multifactorial in origin. Possible contributing factors include the complexity of theatre environments, the urgency of the crisis, medication errors and psychophysiological variables that are unique to each individual and team.^4,6,7^

Framing errors can be divided into two distinct types: equivalence framing and emphasis framing. Equivalence framing is the preference to behave differently depending on whether a decision is viewed as a gain or a loss. This ‘framing effect’ was first characterised by Tversky and Kahneman in 1981.^8^ Emphasis framing is when your subsequent thinking is swayed by the leading aspect of initial presentation. Therefore, decisions involving life-threatening conditions could potentially be influenced by the way in which medical information is framed.^9^ We postulated that the initial framing of a scenario may result in error through a cognitive error called ‘fixation error’. Fixation errors have also been called ‘anchoring errors’ or ‘tunnel vision’ and can be broadly considered as human errors of insight.^2^ Fixation errors are a type of cognitive error in which individuals and teams focus on one aspect of a situation while ignoring more relevant information.^6,7,10,11^

We were unable to demonstrate objective evidence for these cognitive errors in our simulation. Examiners noted that participants did seem to fixate on a single diagnosis (like bronchospasm), but that it appeared to occur regardless of the cognitive trap. We did not power this study to examine each secondary outcome, but there were non-significant differences that might inform the design of future studies in this area. For example, the experimental group appeared quicker to auscultate the chest, which makes intuitive sense when bronchospasm is the primary consideration. They also took longer to respond by increasing the FiO_2_ and suctioning the ETT with a flexible suction catheter. This would be an understandable reaction when managing acute bronchospasm, as opposed to a mucous plug. Despite the delayed response to suctioning down the ETT, the response time to removing the ETT was similar in both groups. This may possibly highlight the reluctance of the candidates to remove the ETT, irrespective of the clinical scenario. It appears that the experimental group marginally diagnosed bronchospasm more frequently and was less likely to entertain the correct diagnosis. However, this remains speculative in the absence of a larger study. Future studies can use our findings to perform sample size calculations for studies aimed at investigating these important questions. Understanding these errors is important in order to find strategies that minimise human error and ultimately improve patient outcomes. Overall, only 38% of the candidates performed the intervention (removing the ETT tube) that would have rectified the situation and saved the patient’s life. This included seven participants in the control group and six in the experimental group.

The participants rated the experience highly with regard to relevance, realism and quality. This positive experience of the simulation was ranked higher than expected by the investigator and examiners, as it was felt that some participants looked emotionally affected by the experience. This may be partly because of the lack of simulation training and experience in the Pietermaritzburg Complex and may change the way that future simulation scenarios are handled. Performing a similar study in an institution that routinely practices simulations would provide an interesting platform for comparison. During feedback, more people in the experimental group stated that they felt misled by the handover. Importantly, they were able to recognise the cognitive trap after the simulation. However, even though they held bronchospasm as an important consideration, they were able to perform in a similar manner to the control group. This recognition post simulation points to the importance and possible benefit of using similar simulations as cognitive aids to recognise our own errors in crisis situations.

### Limitations

This was a single study in an academic centre that does not routinely incorporate simulation as part of the routine curriculum. Unfamiliarity with simulation may have influenced our results. In addition, the data collection took over a year and a half, which created the potential for details of the simulation to have been discussed within the anaesthetic department and thereby introduce performance bias. Further, it is possible that, on observing the performance of candidates during the first round, the teaching of future participants was altered to incorporate routine algorithmic approaches to hypoxia under anaesthesia.

### Strengths

The study design and methodology were prospective, randomised and double blinded, which reduced the possibility of confounding errors. We further standardised the level of training by recruiting participants who had completed the DA. We have demonstrated that this type of study is possible within an academic department, and our results should be able to inform and assist with the design of future studies in this important and understudied area.

### Future research

Similar studies could aim to explore cognitive biases at different levels of training, including undergraduates, interns, medical officers, registrars and specialists. Cognitive biases are likely to be different in expression at different levels of experience. Increasing sample size of studies could be achieved by including disciplines involved in acute care, such as the Emergency Department and the intensive care unit. Simulation-based training creates a platform for anaesthetists to practice and improve their decision-making skills and correct cognitive biases.^12^ The use of simulation-based training and cognitive aids can potentially contribute to safer and more effective anaesthesia care.^13^ Further research is warranted to explore the existence of and evaluate the effectiveness of interventions aimed at reducing cognitive biases.

## Conclusion

Our study showed that the framing of a clinical handover did not result in a difference in time to correct diagnosis in a simulated emergency scenario. Differences in secondary outcomes between groups require investigation in larger studies. Candidates did poorly overall, with only a third of participants correctly resolving the clinical scenario. Simulation training and research provide a useful tool to evaluate cognitive errors and to provide training in scenarios where these errors may occur.
